# The Dynamic Role of NK Cells in Liver Cancers: Role in HCC and HBV Associated HCC and Its Therapeutic Implications

**DOI:** 10.3389/fimmu.2022.887186

**Published:** 2022-05-20

**Authors:** Muhammad Sajid, Lianxin Liu, Cheng Sun

**Affiliations:** ^1^ Department of Hepatobiliary Surgery, Anhui Provincial Clinical Research Center for Hepatobiliary Diseases, Anhui Province Key Laboratory of Hepatopancreatobiliary Surgery, The First Affiliated Hospital of University of Science and Technology of China (USTC), Division of Life Sciences and Medicine, University of Science and Technology of China, Heifei, China; ^2^ Transplant and Immunology Laboratory, The First Affiliated Hospital of University of Science and Technology of China (USTC), Division of Life Sciences and Medicine, University of Science and Technology of China, Hefei, China; ^3^ Chinese Academy of Sciences (CAS) Key Laboratory of Innate Immunity and Chronic Disease, School of Basic Medical Sciences, Division of Life Sciences and Medicine, University of Science and Technology of China, Hefei, China; ^4^ Institute of Immunology, University of Science and Technology of China, Hefei, China

**Keywords:** HCC, NK cells, innate immune landscape, tumor immune microenvironment, HBV induced HCC

## Abstract

Hepatocellular carcinoma (HCC) remains an important complication of chronic liver disease, especially when cirrhosis occurs. Existing treatment strategies include surgery, loco-regional techniques, and chemotherapy. Natural killer cells are distinctive cytotoxic lymphocytes that play a vital role in fighting tumors and infections. As an important constituent of the innate immune system against cancer, phenotypic and functional deviations of NK cells have been demonstrated in HCC patients who also exhibit perturbation of the NK-activating receptor/ligand axis. The rate of recurrence of tumor-infiltrating and circulating NK cells are positively associated with survival benefits in HCC and have prognostic significance, suggesting that NK cell dysfunction is closely related to HCC progression. NK cells are the first-line effector cells of viral hepatitis and play a significant role by directly clearing virus-infected cells or by activating antigen-specific T cells by producing IFN-γ. In addition, chimeric antigen receptor (CAR) engineered NK cells suggest an exclusive opportunity to produce CAR-NKs with several specificities with fewer side effects. In the present review, we comprehensively discuss the innate immune landscape of the liver, particularly NK cells, and the impact of tumor immune microenvironment (TIME) on the function of NK cells and the biological function of HCC. Furthermore, the role of NK cells in HCC and HBV-induced HCC has also been comprehensively elaborated. We also elaborate on available NK cell-based immunotherapeutic approaches in HCC treatment and summarize current advancements in the treatment of HCC. This review will facilitate researchers to understand the importance of the innate immune landscape of NK cells and lead to devising innovative immunotherapeutic strategies for the systematic treatment of HCC.

## Introduction

Primary liver cancer is the fifth most common cancer with high incidence and prevalence throughout the world. Epidemiological data revealed that liver cancer is considered the second-highest mortality rate among different types of cancer, accounting for about 9.1% of total cancer deaths (1, 2). The most common liver cancer is hepatocellular carcinoma which accounts for approximately 90% of cancers worldwide and 75% occurring in Asia and it is the 4^th^ leading cause of mortality (3, 4). Several factors are involved in the development of liver cancers, the most important include genetic and environmental factors. Genetic factors include various single nucleotide polymorphisms (SNPs) and some rare monogenic diseases that influence the individual to HCC (5, 6). The most dominant environmental risk factors include chronic infection with hepatitis B or C virus, alcoholic abuse, nonalcoholic fatty liver disease (NAFLD), the metabolic disorders associated with diabetes mellitus, and obesity that portend a huge trajectory in the frequency of HCC progression (7). Due to the widespread metabolic disorders, liver cancer is supposed to be a rapidly growing cause of cancer-associated mortality. A model-based simulation predicts the increase in HCC mortality globally until 2030. The high frequency of HCC incidence emphasizes the tremendous significance of liver cancers (8).

The liver is a vital organ and is involved in an array of the different physiological functions of the body which include glucose storage, metabolism, detoxification, vitamin storage, cholesterol, and lipid homeostasis, blood and immune regulation, and xenobiotics processing (9). The liver is constantly assaulted by different pathogens and must proficiently respond to maintain host survival and integrity. Intricate cellular surveillance approaches have been developed that facilitate the individual cells to distinguish damage and pathogen-associated molecular patterns. The capability of the liver to cope with these insults is manifested by its specific immune environment. The liver contains the tissue-resident macrophages called Kupffer cells, tissue-resident lymphocytes known as natural killer (NK) cells, NKT cells, conventional and unconventional immune cells namely αβ T cells, γδ T cells, and B cells which makes the liver an immunotolerant organ that limits the progression of chronic liver diseases such as liver cancers and cirrhosis (10). The conventional NK cells (cNKs) and tissue-resident innate lymphoid cells 1 (trILC1s) control the metastatic load in the liver but do not appear to have redundant effects. The cNKs and trILC1 cooperate in a non-redundant manner to control liver metastasis and the metastatic niche affects each innate subset differently in a cancer-type-specific manner (11). Thus, understanding the physiology of the liver is a prerequisite to understanding the pathological or immunological condition of the HCC patients.

**Figure 1:**
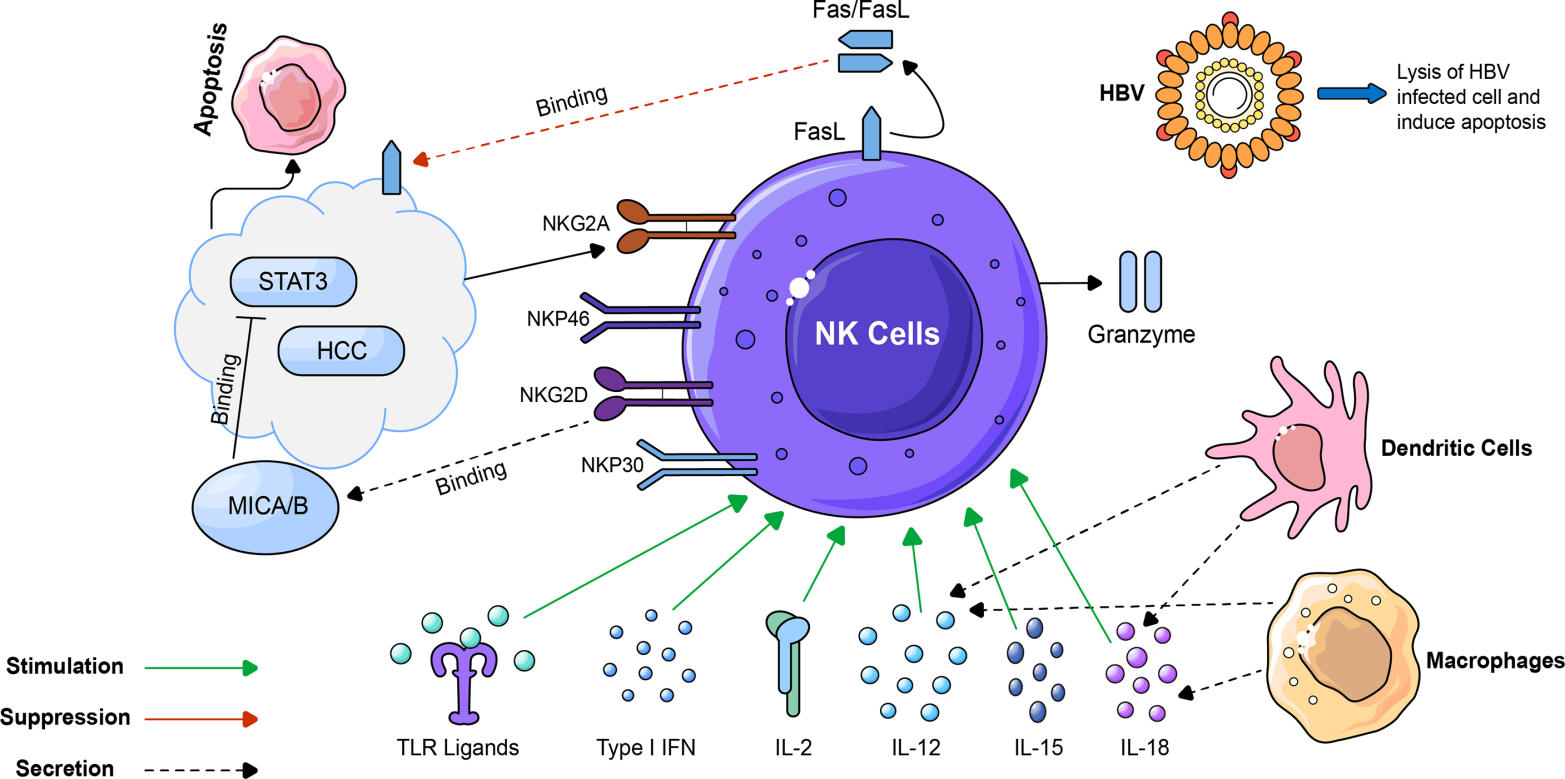
The general overview of the role of NK cells in HCC and HBV-induced HCC. An overview of important cells, cytokines, ligands, and their receptors involved in HCC growth and development. As an important part of our immune system, NK cells play an important role in different stages of HCC development. Their functions are mainly regulated by interactions with other immune cells such as macrophages and dendritic cells, which are mediated by different types of cytokines, ligands, and their receptors. Macrophages and dendritic cells stimulate NK cells by secreting pro-inflammatory cytokines such as IL-12, IL-15, and IL-18. Together with the above-mentioned cytokines and ligands, IL-2, type I IFN, and TLR ligands further stimulate and increase NK cell cytotoxicity. Upon activation, NK cells secrete pro-inflammatory cytokines and induce hepatic stellate cell apoptosis and HBV lysis using Fas/FasL and granzyme/perforin-mediated mechanisms. NKG2D is a significantly important receptor that is activated upon binding to its ligand MICA/B and subsequently leads to enhancing the cytotoxicity of NK cells and blocking STAT3. All of these lead to apoptosis of HCC cells and prevent HCC progression and metastasis.

In humans, extrahepatic and intrahepatic cells, as the main cells of our innate immune system, play a significant role in the body’s immune response to cells infected with different viruses such as HBV or HCV, as well as tumors such as HCC. These NK cells have multiple functions such as granzyme/perforin-mediated apoptosis, Fas/Fasl-mediated cell death, production and secretion of different types of cytokines, and cytokine activation of NK and cytotoxic T lymphocytes (**Figure 1**) (12, 13). All extrahepatic and intrahepatic cells communicate with each other in a coordinated manner to keep the normal homeostasis of the immune system.

Despite the availability of advanced therapeutic approaches and exhaustive research activities, most HCC patients still face a miserable HCC prognosis (14). Systematic palliative treatment is required for these patients which are comprised of tyrosine kinase inhibitors (TKI) such as sorafenib, regorafenib, and lenvatinib, and are employed as a first-line treatment option for HCC patients. Similarly, a human monoclonal antibody such as cabozantinib and ramucirumab explicitly targets vascular endothelial growth factor receptor type 2 (VEGF-R2) and is employed as a second-line treatment option for HCC patients (15–17). However, these systematic treatments yield superficial survival benefits and thus emphasize the need for new therapeutic approaches for HCC treatment (18). The current review will summarize the dynamic role of NK cells in different liver cancers particularly HCC and HBV-associated HCC and its therapeutic implications.

## Natural Killer (NK) Cells as an Active Player in Innate Immune Response

NK cells are the main component of innate lymphoid cells (ILCs) that have a key role in immune defense mechanism, not only as an immunosurveillance, but also to play a cytotoxic role against different viral and transformed infected cells and produce chemokines and cytokines (19, 20). The frequency of NK cells decreases in the blood of Hepatitis B and C viral infection and increases in the liver (21). About 15% of all lymphocytes are comprised of human NK cells and phenotypically defined based on the presence of CD56 expression with the absence of CD3 expression and more than 30% proportion of NK cells solely found in the liver (22, 23). The activity of natural killer cells depends on inhibitory and activating signals communicated through a large number of surface receptors. The killing of healthy cells is prevented by NK cells through different inhibitory receptors, which include KIR, ILT2/CD85, and CD94/NKG2A (24–26). Activating receptors such as DNAM1, NKp46, NKp30, NKp44, and NKG2D identify self-proteins mainly expressed on stress target cells (27). All these receptors coordinate to maintain the normal homeostasis of natural killer cells.

NK cells function primarily by recognizing and killing target cells and secreting cytokines such as interferon-γ (IFN-γ) and tumor necrosis factor-α (TNF-α), which can control antiviral immune responses. NK cells also play a vital role in linking innate and adaptive immune responses by producing antiviral cytokines and chemokines (28). On the basis of CD56 cell membrane density, NK cells can be divided into two subpopulations, CD56^bright^ and CD56^dim^. CD56^dim^ NK cells express high levels of the low-affinity Fc receptors CD16 and killer immunoglobulin-like receptors (KIRs) and account for approximately 90% of all blood NK cells, whereas CD56^bright^ NK cells account for less than 10% of all blood NK cells and do not express CD16 and KIR (29). Functionally, CD56^dim^ NK cells proficiently kill target cells through degranulation but secrete low levels of cytokines; conversely, CD56^bright^ NK cells produce large amounts of cytokines when stimulated but are less cytotoxic. However, CD56^bright^ cells exhibited similar or enhanced cytotoxicity to target cells after prolonged activation compared to CD56^dim^ cells (30). In addition, CD56^bright^ NK cells constitutively express high- and intermediate-affinity interleukin 2 receptors (IL-2R) and increase *in vitro* and *in vivo* in response to low doses of IL-2 (31). On the contrary, resting CD56^dim^ NK cells express only moderate-affinity IL-2R *in vitro* in a weak proliferative response to high doses of IL-2 even after induction of high-affinity IL-2R (32). Therefore, the NK cell is considered as one of the active players in maintaining the innate immune response of the body.

## The Impact of Tumor Immune Microenvironment (TIME) on the Function of NK Cells and Biological Function of HCC

The intrahepatic microenvironment is crucial for NK cell permanency, proliferation, function, and persistence. The HCC microenvironment is a dynamic constituent of the tumor that supports passive structure for tumor development which fluctuates vigorously. Thus, affects the performance of HCC progression. These immunosuppressive landscapes of HCC represent a perplexing hurdle for clinicians to propose operational immunotherapies for liver cancer. The HCC microenvironment is not only comprised of stroma-produced growth factors, metabolites, chemokines and cytokines, and tumor cells, but also tumor-infiltrating macrophages, myeloid-derived suppressor cells, neutrophils, cancer-associated fibroblasts, and regulatory T cells. They all play important roles in the clinical consequence and accomplishment or failure of HCC immunotherapy (23, 33).

**Figure 2:**
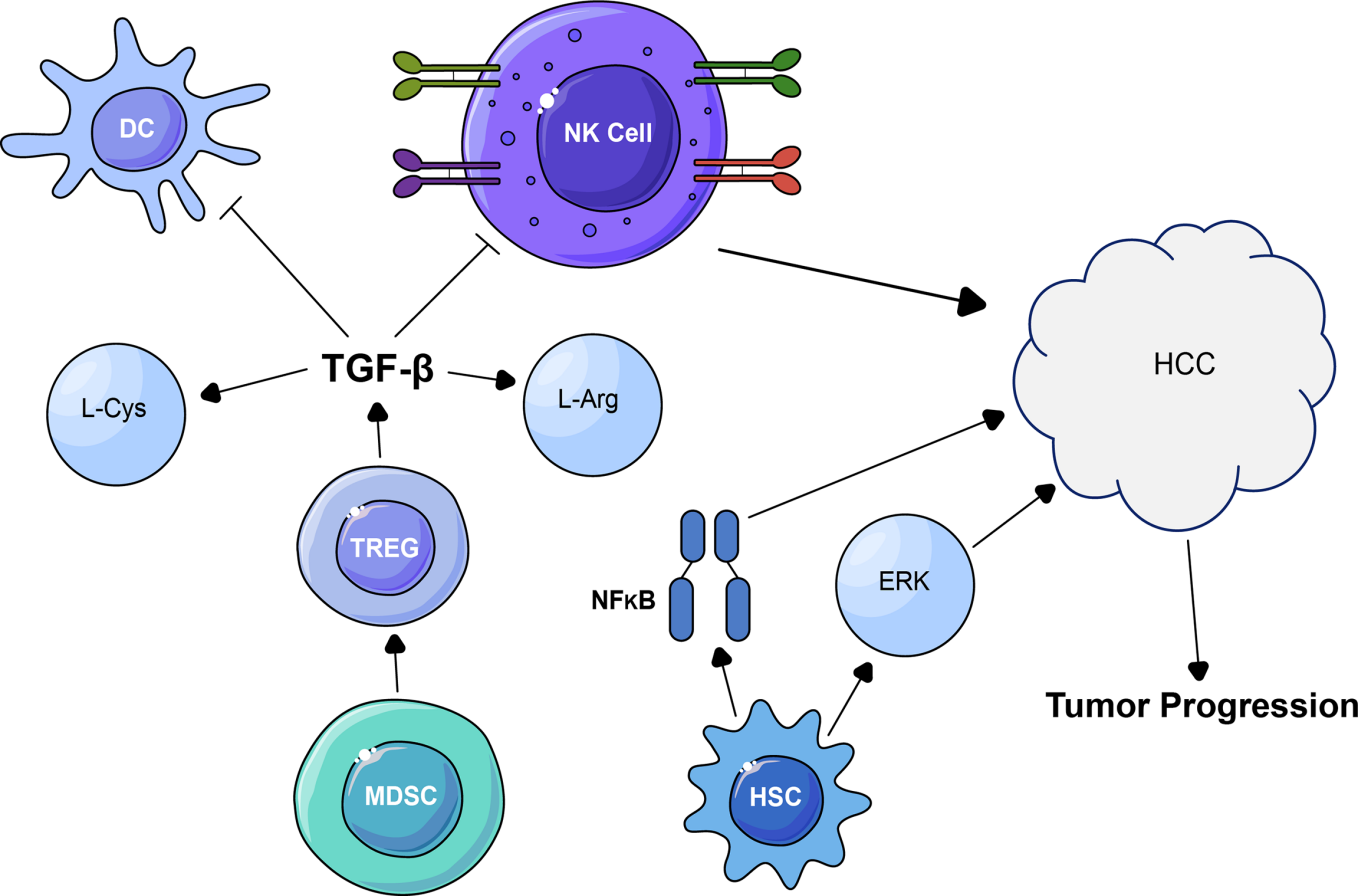
The Impact of Tumor Immune Microenvironment (TIME) on the function of NK cells and the biological function of HCC. MDSCs stimulate differentiation and development of Tregs during tumorigenesis; suppress NK and DCs cells through TGF-β, dispossess amino acids, such as L-cysteine and L-arginine, and produce oxidative stress and the microenvironment becomes hypoxic and leads to HCC progression.

Several immune cells perform various functions in the TIME of HCC. The Myeloid-derived suppressor cells (MDSCs) employ numerous mechanisms of immunosuppressive activity in the TME. MDSCs stimulate differentiation and development of Tregs during tumorigenesis; suppress NK and DCs cells through TGF-β, dispossess T cells of indispensable amino acids, such as L-cysteine and L-arginine, and produce oxidative stress that leads to HCC progression (**Figure 2**) (34, 35). Co-culture of MDSCs with autologous T cells stimulates enlarged Treg numbers, PD-1+ depleted T cells, and increased levels of immunosuppressive cytokines in HCC patients (36). MDSCs also suppressed the production of TLR ligand-stimulated IL-12 and suppressed the T-cell stimulating activity of DCs in HCC (37). MDSCs also weaken NK cell function. In HCC, MDSCs suppress cytotoxicity of NK cells and release cytokine-mediated *via* the NKp30 receptor (38). Tumor-associated neutrophils can recruit Treg cells and macrophages to HCC to stimulate its development and resistance to sorafenib treatment therapy (39, 40). Thus, MDSCs are involved in the regulation of tumor progression.

Hepatic stellate cells (HSCs) are the main skeleton of HCC and can stimulate the proliferation of tumor cells. Conditioned media obtained from HSCs not only induce propagation and migration of HCC cells but also stimulate HCC growth by activating NFκB and extracellular regulated kinase (ERK) pathways (41). Cancer-associated fibroblasts are the predominant cell type within the tumor stroma and play a key role in tumor-stroma interactions (42). They are triggered *via* TGF-b and are liable for the removal, synthesis, and restoration of surplus extracellular matrix, thereby regulating the biological function of HCC. HCC cell extravasation, growth, and metastatic extent depend on the manifestation of these fibroblasts. HCC cells can mutually induce the propagation of tumor-associated fibroblasts, signifying their critical role in tumor-stroma interfaces (43). Stroma derived from HCC expresses numerous growth factors, including epidermal growth factor (EGF), hepatocyte growth factor (HGF), fibroblast growth factor (FGF), stromal-derived factor (SDF)-1a and IL-6, and members of the Wnt family (44). The TIME not only shapes the metabolism of NK cells but is also involved in effector function. Immunometabolic inhibition is critical for designing a new cohort of effective natural killer cell immunotherapies against solid tumors such as HCC. Numerous factors can restrain the metabolism of NK cells in the tumor microenvironment. HCC employs immunosuppressive activity through multiple mechanisms and a well-known driver is a hypoxia (45). The high oxygen depletion of tumor cells produces hypoxic regions. Hypoxia damages effector function of the NK cell but also maintains HIF1a, thereby promoting glycolytic metabolism (46). Hypoxia promotes the production of adenosine on antigen-presenting cells from the cancer-associated extracellular enzymes CD73 and CD39 (expressed on Treg) (47). Tumor cells also produce extracellular adenosine *via* the CD73 and CD39 and exonucleotidases, thereby impairing NK cell function by competing for nutrients (48). Human intratumoral CD96^+^ NK cells are functionally depleted in humans. Patients with higher intratumoral CD96 expression have the poorest clinical outcomes. Obstruction of CD96-CD155 interaction or TGF-β1 reestablishes NK cell immunity to tumors through reversing NK cell depletion, signifying a promising therapeutic role for CD96 in combating liver cancer (49). Therefore, it is important to understand the function of NK cells in the tumor immune microenvironment to fully understand the biological role of HCC.

## The Dynamic Role of NK Cells in Liver Carcinoma

### Role of NK Cells in HCC

HCC is immunologically cold as compared to other liver cancers that are only about 25% of immunoactive phenotypes (50, 51). The number of NK cells in HCC induced individuals is lower within the periphery and functionally inactive. NK cells exist less frequently as compared to myeloid and other lymphocytes within the tumors (52). As compared to peritumoral tissue, HCC accumulation of NK cells in intratumoral tissue has increased expression of CD56^brigh^: CD56^dim^ ratios with enhanced expression of activation marker CD49A show local differences in these two smart microenvironments (53). The frequency and enhanced cytokine secretory function and increased cytotoxicity of NK cells in individuals have enhanced overall survival in HCCs after the liver resection (54, 55). The survival rate of patients is also positively correlated with the occurrence of intratumoral and circulating NK cells in HCC patients, and according to previous studies, the higher the frequency of intratumoral NK cells, the better the response of sorafenib therapy (55–57). A previous study revealed that sorafenib also acts through direct immunomodulation, which is essential for its antitumor activity. *In vivo* and mouse models of HCC identify macrophages (MΦs) as key mediators of antitumor effects and reveal that MΦs and NK cells are strongly interdependent in efficiently killing tumor cells. Additionally, sorafenib was found to downregulate the expression of major histocompatibility complex class-I (MHC-1) of tumor cells which might diminish tumor response to immune checkpoint therapy and favor NK cell response (58).

NK cells perform various functions in HCC related individuals to release cytokines. It was previously considered that IL-6 promotes elliptical cell proliferation and regeneration while TNF-α did not do so. Therefore, the lack of IL-6 results in a significant increase in the development of HCC along with a reduced level of NK cells. This indicates that IL-6 is responsible for NK cell-mediated HCC suppression but has not elucidated a potential mechanism (59–61). IL-1α has a variety of forms *in vivo* and each form has a different function. Membrane IL-1α suppresses the growth of HCC by stimulating T and NK cells while enhancing cytotoxic NK and T cell cytotoxicity (62). Generally, the release of membrane IL-1α and IL-6 increased the protection of HCC through NK cell-related mechanisms (63). In addition, these cytokines may have a vital role in obstacles related to HBV infection, such as HCC and cirrhosis, showing NK cells in the development of HCC (64, 65). Many activated NK cell receptors are related to HCC. Differential splicing in NKP30 results to generate suppressive isoforms that prevail in advanced HCC (66). However, most of the research has focused on NKG2D, which involves a number of ligands elements including UL16-binding proteins (ULBPs) and MHC-I related chain molecule A and B (MICA/B) proteins. These stress-induced proteins are expressed on tumors and can be secreted into the circulation by subsequent proteolytic cleavage, through molecules comprising ADAM9, which is upregulated in HCC (67). Soluble receptors found on the surface of NK cells bind and involve in the downregulation of NKG2D, therefore, making the cells less active. In HCC, high levels of soluble ULBP1 are related to poor survival (68). NKG2D can drive tumors in HCC mouse models, which may promote the intricacy of the molecular pathology of this disease by promoting mutagenic chronic inflammation. However, the downregulation of the NKG2D ligand axis ratio is related to the poor outcome of the disease (69, 70). Therefore, NK cells play an important role in pathogenesis of HCC.

### Role of NK Cells in HBV Induced HCC

NK cells play a vital role in HBV-induced HCC. HBV acts as a stealth virus inside the hepatocytes and NK cells are used as two-edged swords of chronic HBV infection (71). NK cells are hepatoprotective and anti-fibrotic, while activation of NK cells arbitrates HBV-related hepatocyte injury. Recent studies have shown that IFN-γ derived from NK cells induced HCC through the epithelial cell adhesion molecule–epithelial-to-mesenchymal transition axis in HBV transgenic mice, and hepatitis B surface antigen (HBsAg)-positive hepatocyte damage was facilitated through activated NK cells, which stimulated the HCC development (72, 73). NK cells are related to early anti-tumor and antiviral properties (74). Inflammatory cells yield diverse cytokines and chemokines that regulate HBV-induced HCC tumors (**Figure 1**). The specific blocking of interleukin (IL)-10 and transforming growth factor (TGF)-β resumed an immunosuppressive environment in patients with chronic HBV infection by reducing anti-viral efficacy of NK cells (75, 76). Transmission of HBV in NK cells is regulated through HBV positive exosomes, which induces damage to NK cell function, including production of interferon (IFN) -γ, cell solubility, proliferation, and survival of NK cells (77). Further, the expression of miRNA-146A was increased in NK cells from HBV-induced HCC patients as compared to healthy individuals. The cytotoxicity of NK cells and production of IFN-γ and TNF-α of HBV-induced HCC patients were reduced as compared to healthy individuals (78). Therefore, NK cells along with different cytokines and chemokines play a significant role in HBV induced HCC.

In HBV-induced HCC, NK cells have always played a differing role because they employ antiviral and immunomodulatory functions while contributing to the pathogenesis of liver injury. The apoptosis of HBV-infected hepatocytes is conducive to self-protection because it helps clearance of virus from hepatocytes (79). However, in the case of continuous infection, the sustained injury of the liver is the premise of liver fibrosis and HCC. In the immunoactive stage of chronic hepatitis B (CHB), NK cells are biased towards cytolytic activity, which is positively correlated with the severity of the liver injury. Increased NK cell cytolytic activity can be partially ascribed to the change of certain cytokines and the identification between NKG2D and their ligands (80, 81).

NK cells are responsible to employ antiviral effects *via* perforin and tumor necrosis factor-related apoptosis-inducing ligand (TRAIL) dependent cytolysis of infected cells and stimulation of IFN-γ production (82, 83). Phenotypic heterogeneity of NK cells has been described among HBV-induced HCC patients. It had increased expression levels of activating receptor NKG2D and a reduction in the inhibitory receptor, while the percentage of activating NKG2C+ NK cells amplified and the inhibitory receptor expression was normal in chronic HBV patients (84, 85),. It is believed that NK cells decreased in patients with chronic viral hepatitis patients to promote disease progression and conversion to HCC. In acute HCV infection, hepatic NK cells showed an increase in NKP46 expression and potentially degranulate and stimulate IFN-γ that subsequently activate IFN-α/β and other cytokines (IL-12, IL-15, IL-18) (**Figure 1**) (86, 87). NK cell repertoire in HBV patients co-infected with HCV is influenced towards memory-like phenotype related to enhanced CD16 mediated ADCC effector function. Even though liver NK cells sustain their cytotoxic role in chronic HBV infection through the upregulation of TRAIL, they eradicate autologous HBV specific CD8+ T cells expressing elevated levels of death receptor for TRAIL (88, 89). Thus, NK cells are indispensable to play a vital role in antiviral and immunomodulation in HBV-induced HCC.

## NK Cell-Based Therapeutic Approaches

NK cells, as the main immunomodulatory constituent of our immune system, can directly kill tumor cells without antigen sensitization. A previous study indicated the prospective of cancer immunotherapy to control the activation of NK cells (90). Therefore, directly targeting NK cells can serve as a promising therapeutic strategy that can considerably improve the therapeutic process. (**Table 1**)

**Table 1:** NK Cell based Therapeutic Approaches.

Treatment Approaches		Mechanism	References
Chemotherapeutics	Polysaccharide SEP	Act through TLR2/TLR4 and activate NK cells and T lymphocytes and enhance antitumor activity	(91)
	Gemcitabine (GEM)	the cytolytic activity of NK-92 cells by inhibiting ADAM10 and upregulating MICA expression and reducing sMICA secretion	(92)
	Sunitinib	upregulate of NKG2DLs, through non-canonical pathways of NF-kb signaling, apoptotic, and DNA damage repair genes	(93)
	Muromonab-CD3	together with the causes enhanced natural killer cell activation and T lymphocyte exhaustion	(94)
	Sorafenib	initiates the pro-inflammatory activity of tumor-associated macrophages. stimulation of antitumor natural killer cell responses in a cytokine and NF-kB dependent manner.	(95, 96)
		Activation of Natural killer cell in the hepatic portal system enhance cytotoxicity of IL-12l and IL-12A	
	Regorafenib	multi-kinase inhibitor anticancer drug suppressing c-RAF, c-KIT, RET, TIE-2, PDGFR, BRAF, VEGFR1-3, FGFR-1, and p38 MAP kinases	(97, 98)
CAR-NK Based Therapeutic Approaches	Glypican-3 (GPC3)-specific CAR-NK-92	greater antitumor and cytotoxicity activity against HCC xenografts	(99)
	Basigin	Kills malignant HCC in patient derived tumors and mouse xenograft model	(100)
**iPSC-NK cell Based Therapeutic Approaches**		Enhanced antitumor activity in combination with different cytokines	(101–122)
Immunotherapeutics	TRAIL	inhibit hepatocellular carcinoma *via* enhancing infiltration of NK cell in the tumor microenvironment	(123, 124)
	TLR7 and TLR8 agonists	enhance the efficiency of NK cell-based immune surveillance through stimulating NK-DC crosstalk which lead to type I IFN and IL-12 dependent enhancement of NK cell function, and enhanced antitumor immune response to HCC	(125)
	chimeric NKG2D-CD3ζ-DAP10 receptor	increased the signaling capability of the NKG2D receptor, resulting in increased cytotoxic activity of the expanded natural killer cells	(126)
Antimicrobials and novel treatment	Anisomycin	novel therapeutic HCC drug that controls CD58, ICAM4, lymphocyte-associated antigen 3 and MHC-1, to improve immunity and acts as an immunological synapse between HCC and natural killer cells	(127, 128)
	Lomofungin	antifungal agent that reduces activity of ADAM17 and then reduced sMICA activity in a dose-dependent manner	(129, 130)
	HDACI trichostatin A	enhancing cytotoxicity of NK cell in HCC through transcriptional regulation of RAET1G and ULBP1 and enhance apoptosis of HCC cells	(131)
	Gastrodin	causes increased cytotoxicity of natural killer cells and CD8+ T lymphocytes	(132)
	Mellitin	antitumor, antibacterial, antiarthritic, and anti-inflammatory characteristics.	(133, 134)
	Mel-P15	initiate cytotoxicity of NK cells and potentiates TNF-α, IFN-γ and IL-2 secretion	(135)

## Chemotherapeutic Approaches

A number of chemotherapeutic approaches and agents have been discovered to affect NK cells. The polysaccharide SEP acting through TLR2/4 has been shown to stimulate T lymphocytes and natural killer cells. It also increased the antitumor activity of the pyrimidine antimetabolite gemcitabine (GEM) against HCC by activation of the NKG2D and DAP10/Akt pathways that lead to activation of NK cells. It is also hypothesized that upregulation of NKG2D increases the sensitivity of NK-92 cells to targeting their ligand MHC-I related chain molecule A (MICA) expressed on HepG2 cells. GEM enhances the cytolytic activity of NK-92 cells against HepG2 cells by inhibiting ADAM10 and upregulating MICA expression and reducing soluble MICA (sMICA) secretion (91, 92). Multi-targeted tyrosine kinase inhibitors (MTKIs) facilitate upregulation of NKG2D ligands, sensitizing cancer cells to natural killer cell therapy. However, the exact underlying mechanism remains unknown. Sunitinib is an MTKI shown to escalate the stimulation and cytotoxicity of natural killer cells in the HepG2 cell line. It leads to the upregulation of NKG2DLs through non-canonical pathways of NF-kB signaling, apoptotic, and DNA damage repair genes (93). Muromonab-CD3, an anti-CD3 antibody, together with the alternative GMP-CD3 with similar effects on hepatic monocytes, causes enhanced natural killer cell activation and T lymphocyte exhaustion (94). Administration of sorafenib initiates the pro-inflammatory activity of tumor-associated macrophages by sensitizing them to exogenous immune stimulation. This results in the stimulation of antitumor natural killer cell responses in a cytokine and NF-kB dependent manner. Activation of natural killer cells triggered through sorafenib is dependent on the activity of LPS, an exogenous factor that can be present profusely in the hepatic portal system (95). A previous study reveals that sorafenib may enhance cytotoxicity by suppressing AR, thereby enhancing a subtype of IL-12 and IL-12A (96, 97). Regorafenib is a newly discovered multi-kinase inhibitor anticancer drug for the treatment of advanced HCC and works by suppressing c-RAF, c-KIT, RET, TIE-2, PDGFR, BRAF, VEGFR1-3, FGFR-1, and p38 MAP kinases. Compared with sorafenib, regorafenib is more effective in inducing apoptosis in HCC cells but the toxicity and side effects are nearly the same (98, 136). A combination of different chemotherapeutics agents might be helpful for future treatment regimes.

## CAR-NK Based Therapeutic Approaches

Numerous approaches have been established to expand the targeting and effectiveness of NK cell cytotoxicity against tumor cells using genetic alteration techniques. Strategies employed to generate chimeric antigen receptor-NK (CAR-NK) cells have also been applied that not only increase the efficacy of NK cell therapy but also largely escalate the specificity of NK cell therapy (137, 138). CARs are comprised of a single-chain extracellular variable fragment (scFv) and an intracellular immune cell activation domain (139, 140). Using CAR-T as a prototype model, preliminary studies on NK cells integrated the 4-1BB co-stimulatory domain, which confers greater persistence; however, 2B4 is an NK cell-specific co-stimulatory domain that improves cell function in a number of ways, including increased cytotoxicity, persistence, proliferation, and cytokine secretion (141). Previous studies revealed four different costimulatory domains (DAP10, DAP12, 2B4, and CD137) and four different transmembrane domains (NKp44, NKp46, CD16, and NKG2D) in combination with CD3ζ to enhance NK cell-specific CAR constructs. These studies validate that CARs containing the 2B4 costimulatory domain and NKG2D transmembrane domain mediate improved antitumor activity *in vitro* as well as in the ovarian cancer xenograft model (142). Furthermore, CAR experiments using PB-NK showed that subpopulations responded differently to CAR stimulation, with terminally differentiated NK cells exhibiting the best response. Moreover, this observation was only tested against the anti-CD19 ectodomain. Determining which subpopulations respond best to modifications in other ectodomains requires further investigation (143). This point confirms that the benefits of using multiple CAR-NK cell populations can be produced for the optimization of cancer therapy.

CAR-NK cells are primary NK cells genetically engineered to express a chimeric antigen receptor (CAR), such as glypican-3 (GPC3) or alpha-fetoprotein (AFP), that promotes the binding of relevant antigens in a specific manner. In addition, NK cells express receptors for the Fc portion of immunoglobulins (FcRs) that bind to target cells and mediate cytolysis of NK cell susceptible target cells (i.e., antibody-dependent cell cytotoxicity ADCC) (144). Among genetically altered NK cells, GPC3 specific CAR-NK-92 cells have been reported to have high antitumor activity against HCC xenografts expressing high and low levels of GPC3. The specificity of GPC3 CAR-NK-92 cells was confirmed by the potent cytolytic activity shown in an *in vitro* cytotoxicity assay against GPC3+ HCC cells, whereas no cytotoxicity was observed in GPC3- HCC cells. Therefore, GPC3-specific CAR-NK cells represent a new therapeutic approach for GPC3+ HCC patients (99). NK and T cells are transduced with a CAR that recognizes basigin (the surface marker CD147) that not only kills several malignant HCC cell lines *in vitro* but also kills HCC tumors in xenograft and patient-derived xenograft mouse models (100). These findings represent the therapeutic potential of CD147-CAR-modified immune cells in HCC patients.

## iPSC-NK Cell Based Therapeutic Approaches

Several studies have genetically engineered induced pluripotent stem cells (iPSCs) to generate iPSC-NK cells with *in vivo* persistence and improved expansion. Many of these techniques were first developed and tested in cord blood-NK (CB-NK) cells and peripheral blood-NK (PB-NK) cells and successively transformed into iPSC-derived NK cells. The iPSCs offer a stable platform for a routine genetic alteration process that only needs to be done in a one-time. Once stably engineered iPSC clones are identified, they can be extended and used to generate standardized populations of properly engineered iPSCs derived NK cells (101, 102). The interleukine-15 (IL-15) plays an important role in stimulating NK cell expansion and cytotoxic function (103, 104). Activation of IL-15 was also revealed to alleviate immunosuppression mediated through transforming growth factor (TGF)-b1 released by the TME. These features make manipulation of IL-15 expression an attractive approach to increase the antitumor activity of various NK cell populations without the need to supplement cell-producing cultures with high doses of cytokines (105). A study conducted by Imamura et al. confirmed that expression of a membrane-bound form of IL-15 (mbIL-15) in human PB-NK cells enhances antitumor killing against hematological malignancies and solid tumors by increasing the survival of NK cells and expanded *in vitro* and *in vivo* without additional exogenous cytokines (106). Another method adopted by two different groups to increase the antitumor activity of iPSC-NK and PB-NK cells *in vitro* and *in vivo* using an IL-15 receptor fusion construct consisting of an IL-15 receptor alpha and IL-15 superagonist (IL-15RA/IL15SA), respectively (107, 108). By employing these approaches, the antitumor activity could be enhanced with greater efficacy.

A broader range of effector functions can be achieved by treating NK cells with cytokines (109). In preclinical and clinical studies, the different groups showed that treatment with a cytokine cocktail consisting of IL-12, IL-15, and IL-18 resulted in the development of cytokine-induced memory like-NK (CIML-NK) cells and enhanced interferon-g (IFN-g) generation and cytotoxicity against leukemia cell lines or primary human AML blasts (110, 111). Their Phase I clinical trial resulted in complete remission in 4 of 9 patients (112). The CIML-NK cells further exhibit enhanced IFN-g production, enhanced cytotoxicity, and persistence against ovarian cancer and other malignancies (113, 114). Different bispecific engager molecules are also involved in therapeutic approaches. The blinatumomab is a CD3-CD19 bispecific antibody that binds and transports CD3+ T cells to CD19+ B-cell in acute lymphoblastic leukemia and several groups have developed multivalent targeting molecules that specifically engage in the vicinity of the target tumor NK cells to enhance tumor killing (115). These Bispecific Killer engagers (BiKEs) or Trispecific Killer engagers (TriKEs) are designed to stimulate NK cells that subsequently activate cell surface cell receptors. For instance, engagers targeting NK cells for CD30+ lymphoma, CD133+ colon cancer, CD33+ myelodysplastic syndrome, CLEC12A+ and CD33+ acute myeloid cell (AML) are all in clinical development. A bispecific CD30xCD16 engager direct CB-NK and PB-NK cells to escalate cytotoxicity against CD30+ Lymphoma in *in vitro* and *in vivo* in preclinical studies (116). A CD16xCD33 bispecific engager and TriKE targeting CD16, CD33, and stimulating IL15 improve NK cell killing of CD33+ myelodysplastic syndrome cells (117, 118). NK cells more efficiently kill CD133+ or EPCAM+ colon cancer cells *via* CD16xCD133 or CD16xEpcam TriKE containing an IL-15 cross-linker (119, 120). The BiKEs and TriKEs targeting CD33 and CLEC2A on AML increased NK cell-mediated killing of CD33+ or CLEC2A+ AML cells, respectively, in preclinical models of AML (121, 122). Clinical trials employing these engineered iPSC-NK cells are underway that could be a benefit in near future.

## Immunotherapeutic Approaches

TRAIL is immunoprotective by enhancing the infiltration of natural killer cells in the tumor environment. TRAIL-expressing NK cells are characteristically reduced by decreased expression of the CXCR3 ligand CXCL9 after hepatectomy. Both hepatectomy and partial hepatectomy lead to downregulation of the CXCL9-CXCR3 axis, resulting in a reduction of TRAIL-expressing NK cells, which is associated with poor prognosis and early HCC recurrence (123). However, new studies have also revealed the combination of oncolytic adenoviruses encoding IL-12 and TRAIL genes can inhibit hepatocellular carcinoma *via* enhancing the infiltration of NK cells in the tumor microenvironment. Therefore, stimulation of TRAIL after hepatectomy to improve diagnostic outcomes has great therapeutic potential (124). Different cytokines agonists such as TLR7 and TLR8 agonists can also enhance the efficiency of NK cell-based immune surveillance through stimulating NK-DC crosstalk. DCs also lead to type I IFN and IL-12 dependent enhancement of NK cell function, resulting in an enhanced antitumor immune response to HCC. A previous study revealed that GPC3-specific chimeric AR-engineered NK cells have been developed with high cytotoxic activity as an effective immunotherapy option for GPC3+ HCC patients (99, 125). Peripheral blood natural killer cells can be extended and activated by using different types of proinflammatory cytokines such as IL-12, IL-21, IL-15, IL-2, and type I IFN (64). It was demonstrated previously that natural killer cells generally had higher levels of cytotoxicity against HCC cell lines than unstimulated or IL-2-activated NK cells. Furthermore, experiments showed that the use of the chimeric NKG2D-CD3ζ-DAP10 receptor increased the signaling capability of the NKG2D receptor, resulting in the increased cytotoxic activity of the expanded natural killer cells *in vitro* and an immunodeficient mouse model (126). Immunotherapeutic agents with the combination of different cytokines agonists can be helpful in enhancing the efficiency of NK cell-based immune surveillance.

## Antimicrobials and Novel Treatments

Recent studies have revealed the possibility of using existing antibacterial agents to treat HCC. Anisomycin is an antibiotic and an inhibitor of eukaryotic protein synthesis produced by *Streptomyces griseolus*. It has shown potential as a novel therapeutic HCC drug in the previous study, as it controls a wide range of genes, such as CD58, ICAM4, lymphocyte-associated antigen 3, and MHC-1 to improve immunity and acts as an immunological synapse between HCC and natural killer cells (127, 128). Lomofungin is an FDA-approved antifungal agent that reduces the activity of ADAM17 and then reduces sMICA activity in a dose-dependent manner (129, 130). The antifungal drug histone deacetylase inhibitors (HDACI) trichostatin A indirectly kills HCC cells by enhancing cytotoxicity of NK cells in HCC through transcriptional regulation of important genes such as RAET1G and ULBP1 leads to directly enhancing apoptosis of HCC cells (131). Thus, existing antimicrobial agents have shown promising potential in the systematic treatment of HCC.

Some novel treatment has been proposed for the treatment of HCC. Gastrodin is a compound extracted from the famous East Asian nourishing food Gastrodiaelata Blume. It can ease the low-toxic growth of transplanted H22 liver tumor cells in mice. Gastrodin causes increased cytotoxicity of natural killer cells and CD8+ T lymphocytes through a mechanism that is poorly understood (132). Mellitin has potential antitumor, antibacterial, antiarthritic, and anti-inflammatory characteristics usually isolated from bee venom. The TT-1, a synthetic novel peptide analog, was developed with more antitumor effects. The combination of IFN-α and TT-1 enhanced the activity of NK cells against HCC cells by stimulating interaction between MICA and NKG2D (133, 134). Another melittin analog, Mel-P15, was also considered to have potential in HCC treatment. It directly initiates cytotoxicity of NK cells and potentiates TNF-α, IFN-γ, and IL-2 secretion (135). Therefore, different antimicrobial agents and novel treatment regimens could be employed for HCC treatment.

## Conclusion

NK cells play a vital role in HCC and inhibit the development of liver fibrosis, cirrhosis, and hepatocarcinogenesis. NK cell-based anti-HCC therapeutic approaches are becoming increasingly attractive. However, most attempts are being explored in animal models or ongoing human clinical trials. The evaluation of long-term antitumor effect and safety requires further observation. A deeper understanding of the mechanisms by which NK cells recognize tumors and HCC evade NK cells in a liver-specific microenvironment will provide valuable insights into the treatment of HCC. The selection of NK cells with predominantly activating properties is critical to providing successful anti-HCC activity. Immunotherapy based on specific NK subsets, especially liver-specific NK cells, may be attractive for HCC patients. Importantly, the strategy of combining NK cell-based immunotherapy with conventional chemotherapy or other multiple therapies will offer greater hope for persistent patients with HCC and HBV-induced HCC.

## Author Contributions

CS conceived and directed the project. MS and CS drafted the manuscript. All authors contributed to the article and approved the submitted version.

## Funding

This work was supported by the National Key Research and Development Program of China (# 2021YFC2300600), National Natural Science Foundation of China (# 8202290021, # 92169118, #91942310) and Anhui Provincial Natural Science Foundation (# 2008085J35).

## Conflict of Interest

The authors declare that the research was conducted in the absence of any commercial or financial relationships that could be construed as a potential conflict of interest.

## Publisher’s Note

All claims expressed in this article are solely those of the authors and do not necessarily represent those of their affiliated organizations, or those of the publisher, the editors and the reviewers. Any product that may be evaluated in this article, or claim that may be made by its manufacturer, is not guaranteed or endorsed by the publisher.
